# Synergy of antioxidant and M2 polarization in polyphenol‐modified konjac glucomannan dressing for remodeling wound healing microenvironment

**DOI:** 10.1002/btm2.10398

**Published:** 2022-09-05

**Authors:** Huiyang Li, Xiaoyu Liang, Youlu Chen, Kaijing Liu, Xue Fu, Chuangnian Zhang, Xiaoli Wang, Jing Yang

**Affiliations:** ^1^ Tianjin Key Laboratory of Biomaterial Research Institute of Biomedical Engineering, Chinese Academy of Medical Science & Peking Union Medical College Tianjin China

**Keywords:** bioactive dressing, M2 macrophage, ROS scavenging, wound healing

## Abstract

Effective skin wound healing and tissue regeneration remain a challenge. Excessive/chronic inflammation inhibits wound healing, leading to scar formation. Herein, we report a wound dressing composed of KGM‐GA based on the natural substances konjac glucomannan (KGM) and gallic acid (GA) that accelerates wound healing without any additional drugs. An in vitro study showed that KGM‐GA could not only stimulate macrophage polarization to the anti‐inflammatory M2 phenotype but also decrease reactive oxygen species (ROS) levels, indicating excellent anti‐inflammatory properties. Moreover, in vivo studies of skin wounds demonstrated that the KGM‐GA dressing significantly improved wound healing by accelerating wound closure, collagen deposition, and angiogenesis. In addition, it was observed that KGM‐GA regulated M2 polarization, reducing the production of intracellular ROS in the wound microenvironment, which was consistent with the in vitro experiments. Therefore, this study designed a multifunctional biomaterial with biological activity, providing a novel dressing for wound healing.

## 1 INTRODUCTION

Skin tissue is the natural barrier that protects the body from environmental damage and microbial infestation.^1^ When the skin is damaged, pathogens are more likely to invade the body and cause inflammation and infection, affecting the process of wound healing.^2^ Currently, several treatments, including growth factors^3^, ^4^, ^5^ and stem cells,^6^, ^7^ can drive wound healing, but these approaches are limited by their high cost and side effects.^8^ However, there have been few studies on regulating the wound microenvironment to utilize the inherent regenerative capacity of the host. Most treatment methods mainly focus on the process of structural repair, and nonfibrotic healing of damaged tissues is still difficult to achieve.^9^ Effective restoration of wound tissue integrity and function remains a global health issue.

Macrophages, which play an important role in tissue repair, are highly plastic cells and can be polarized into classic M1 type (inflammatory phenotype in the early stage) and M2 type (anti‐inflammatory phenotype in the mid‐stage), and these cells can shift between phenotypes under certain conditions.^10^, ^11^ M2 macrophages have been shown to secrete a range of anti‐inflammatory factors, such as interleukin‐10 (IL‐10) and transforming growth factor‐β (TGF‐β), and secrete healing factors that reduce the inflammatory response, promote angiogenesis, and create a regenerative microenvironment.^12^ Appropriate regulation of the M1‐to‐M2 shift is critical in promoting tissue repair and coordinating skin healing. Studies have confirmed that cytokines, such as interleukin‐4 (IL‐4) and interleukin‐13 (IL‐13),^13^, ^14^ can be used to polarize M2 macrophages. However, these recombinant proteins are unstable in the body and difficult to effectively deliver, and excessive use may cause serious side effects, thus limiting their application. Konjac glucomannan (KGM) is a natural polysaccharide that consists of d‐glucose and d‐mannose linked by a β‐1,4 glycosidic chain.^15^ The abundant carbohydrate receptors expressed on the surface of macrophages interact with these sugar units to activate murine monocytes/macrophages, such as the mannose receptor (MR), which responds to mannan.^16^ In addition, KGM has also been reported to be nontoxic, biodegradable, and biocompatible.^17^ Moreover, KGM exhibits outstanding liquid adsorption capacity owing to its structure, which contains plentiful hydroxyl and carboxyl groups that can attract water molecules through hydrogen bonding and van der Waals forces.^18^ These excellent properties of KGM are conducive to its application in wound healing.

In addition to regulating macrophage phenotype to resist inflammation, controlling the level of reactive oxygen species (ROS) in impaired wounds has become another research direction. Many studies have shown that ROS play an important role in the wound microenvironment.^19^, ^20^, ^21^ After skin injury, the wound surface produces abundant ROS, which is one of the defense mechanisms against bacterial infections.^22^ However, high levels of ROS, such as hydrogen peroxide (H_2_O_2_), can cause oxidative stress in the impaired wound and trigger a series of harmful effects, such as cell aging,^23^ fibrotic scarring,^19^ and inflammation.^24^ In addition, ROS can significantly limit angiogenesis, leading to endothelial dysfunction.^20^ ROS can also inhibit the function of endogenous stem cells and macrophages, hinder the regeneration of wound tissue, and ultimately impede the process of wound healing.^22^, ^25^ Therefore, studying biomaterials that have the ability to eliminate ROS and control oxidative damage in the microenvironment of impaired wounds is a potential treatment strategy to promote wound healing. Gallic acid (GA) belongs to a class of natural polyphenol compounds that are widely found in the plant kingdom.^26^ GA has potential anti‐inflammatory and antioxidant effects and can directly upregulate the expression of antioxidant genes. Previously, GA was used in traditional medicines for the treatment of various chronic skin diseases, such as psoriasis and vitiligo.^27^, ^28^ Recent studies have shown that GA promotes wound healing and accelerates the migration of keratinocytes and fibroblasts.^29^ In addition, due to its excellent antioxidant properties and lack of toxicity, GA can be used to modify functional materials to improve their physical, chemical, and biological properties.^30^

**SCHEME 1:**
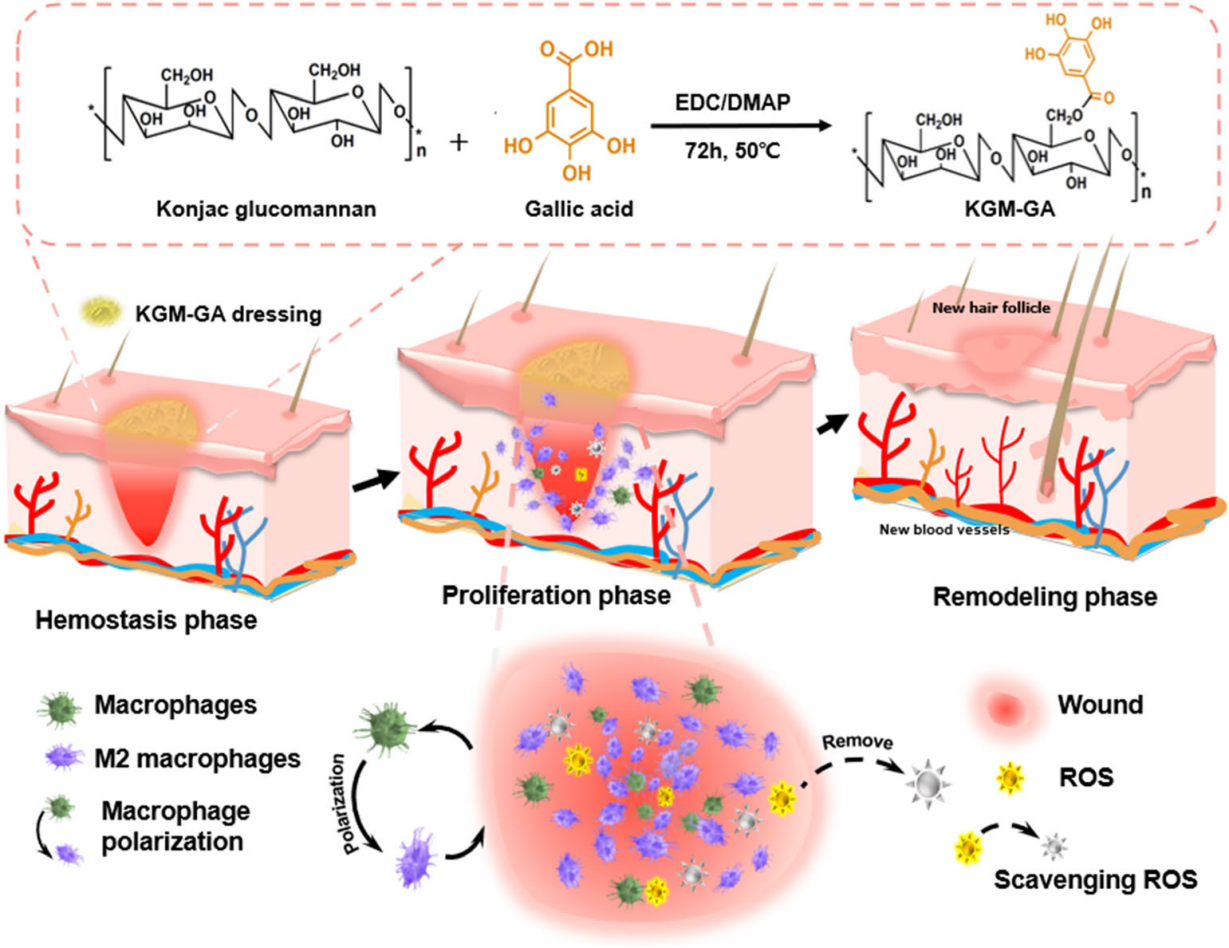
Schematic illustration showing the synthesis of konjac glucomannan–gallic acid (KGM‐GA) and KGM‐GA dressing for accelerating wound healing through M2 macrophage polarization and scavenging reactive oxygen species (ROS).

This study prepared a polymer dressing designed to improve the wound microenvironment, which could regulate the conversion of macrophages to the M2 phenotype while controlling the level of ROS in the impaired wound (Scheme 1). In this study, KGM was conjugated with GA by esterification to prepare a material for skin repair. Our research results showed that in the absence of any exogenous cytokines or drugs, KGM‐GA could significantly upregulate M2 macrophage polarization and eliminate excess ROS, which may provide insight into the development of efficient dressings for improving the wound healing process.

## 2 RESULTS

### 2.1 KGM‐GA synthesis and structural characterization

**FIGURE 1:**
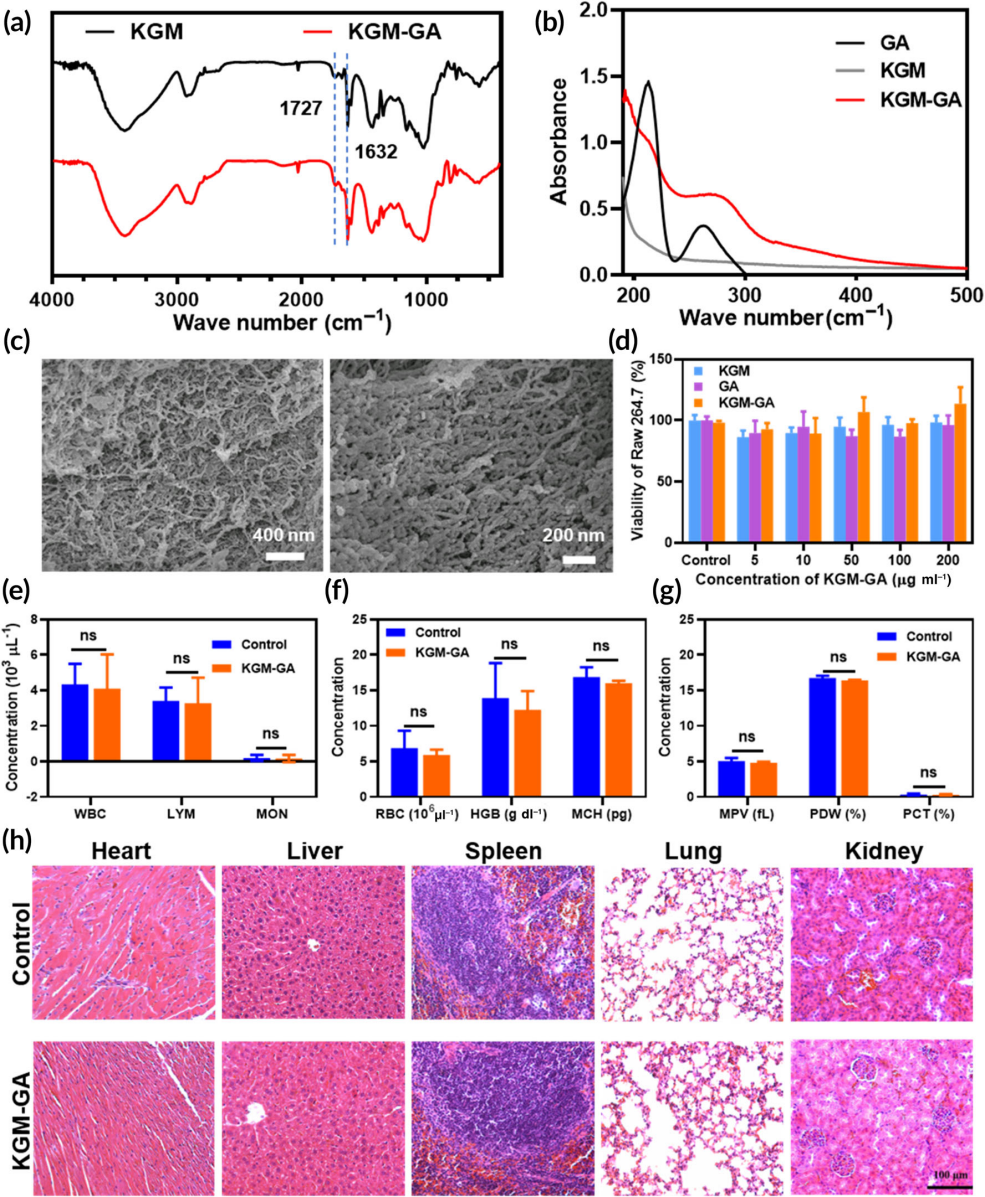
Structural characterization and biocompatibility assessments. (a) Fourier transform infrared (FTIR) spectrum of konjac glucomannan (KGM) and KGM‐GA. (b) UV–vis spectra of gallic acid (GA), KGM, and KGM‐GA. (c) Scanning electron microscopy (SEM) image of KGM‐GA. (d) Viability of Raw 264.7 cells treated with KGM, GA, and KGM‐GA, respectively, for 24 h at different concentrations. (e–g) Blood parameters in normal mice (control group), and mice treated with KGM‐GA after 14 days. (h) Evaluation of in vivo toxicity of KGM‐GA to major organs (heart, liver, spleen, lung, and kidney) at 14 days after treatment compared with normal mice (control group). The data were conducted by two‐way ANOVA with Sidak's post hoc test. The data are represented by means ± SD (*n* = 3, ^ns^ *p* > 0.05).

The reaction of KGM and GA occurred between the hydroxyl group of KGM and the carboxylic group of GA to form the ester linkage using the EDC/DMAP‐mediated coupling reaction. The procedure to synthesize KGM‐GA is illustrated in Scheme 1. The structure of KGM‐GA was first confirmed by Fourier transform infrared (FTIR). Figure 1a shows the FTIR spectra of KGM and GA‐KGM. In the FTIR spectrum of KGM, the absorption band of the carbonyl of the acetyl groups was observed at 1727 cm^−1^. The intense peak at 1632 cm^−1^ was attributed to the in‐plane deformation of the water molecule. In contrast to the spectrum of KGM, the band at approximately 1727 cm^−1^ was also ascribed to the carbonyl, which changed from a small shoulder in pure KGM to a distinct peak in KGM‐GA. The spectrum of KGM‐GA clearly demonstrated successful chemical conjugation. The UV–vis spectra of GA, KGM, and KGM‐GA are shown in Figure 1b. We observed no absorbance peak of the KGM solution within the detection range. In contrast, the KGM‐GA solution showed evident absorbance peaks at 215 and 263 nm, which also appeared in the UV–vis spectra of GA. These results indicate that KGM‐GA was successfully synthesized. The conjugation percentage was calculated by determining the amount of GA conjugated in KGM‐GA according to the standard curve at 263 nm, and the content of GA was determined to be 3% KGM‐GA. Scanning electron microscopy (SEM) images showed that KGM‐GA has a loose, high‐porosity structure (Figure 1c), which was conducive to air permeability and promoted the transfer of nutrients.

### 2.2 Biocompatibility of KGM‐GA

The cytotoxicity of KGM, GA, and KGM‐GA toward Raw 264.7 and L929 cells was evaluated using CCK‐8 and live/dead cell staining assays. As shown in Figure 1d and Figure S1, within a certain concentration range, none of the groups showed obvious cytotoxicity, and the viability of Raw 264.7 cells exceeded 80% and was 95% for L929 cells. Moreover, cell viability was examined using live and dead cell staining in the KGM‐GA group (Figure S2). The majority of cells were green, and at the test concentrations, there was no noticeable cytotoxicity. Next, the effect of KGM‐GA on the hemolysis of red blood cells was studied. As shown in Figure S3, 1 mg/ml KGM‐GA affected the integrity of erythrocytes in the same way as the control PBS buffer (negative group), indicating good biocompatibility.

The in vivo biological safety of a material is a key factor in its application. Next, we evaluated the effects of KGM‐GA on mouse blood chemistry and major organ histopathology to determine in vivo biocompatibility. As shown in Figure 1e–g, complete blood panel analysis showed no obvious differences in hematology at 14 days after treatment with KGM‐GA in wounded mice compared to that of the control (*p* > 0.05). Moreover, no necrosis, congestion, or hemorrhage was observed in the heart, liver, spleen, lung, or kidney (Figure 1h). These results demonstrated the excellent biocompatibility of KGM‐GA in vivo.

### 2.3 Regulation of macrophage polarization in vitro

Macrophages are essential regulators of wound healing and are known to have many functions, including participating in inflammation and promoting tissue repair and regeneration.^31^ According to their activation states and functions, they can be divided into M1 and M2 phenotypes, which play different roles in wound healing. Despite M1 cells playing a critical role in defending against external pathogens in the early stage,^32^ chronic M1 activation and continuous secretion of proinflammatory cytokines delay wound healing.^33^ In contrast, M2 macrophages can secrete anti‐inflammatory cytokines and extracellular matrix components, which are necessary for the late stage of tissue repair,^34^ and studies have shown that the infiltration of M2 macrophages reduces scar formation.^35^

First, to evaluate the effects of different concentrations of KGM‐GA on the polarization of macrophages, we performed flow cytometric analysis of the M1 marker CD86 and M2 marker CD206 in Raw 264.7 cells. As shown in Figure S4, the expression of CD206 increased in response to KGM‐GA in a concentration‐dependent manner. However, at a concentration of 100 μg/ml, the expression of CD86 also increased significantly. Then, we calculated the ratio of CD206 to CD86 and found that the maximum occurred at a concentration of 50 μg/ml KGM‐GA, which was used in subsequent experiments (Figure S5).

**FIGURE 2:**
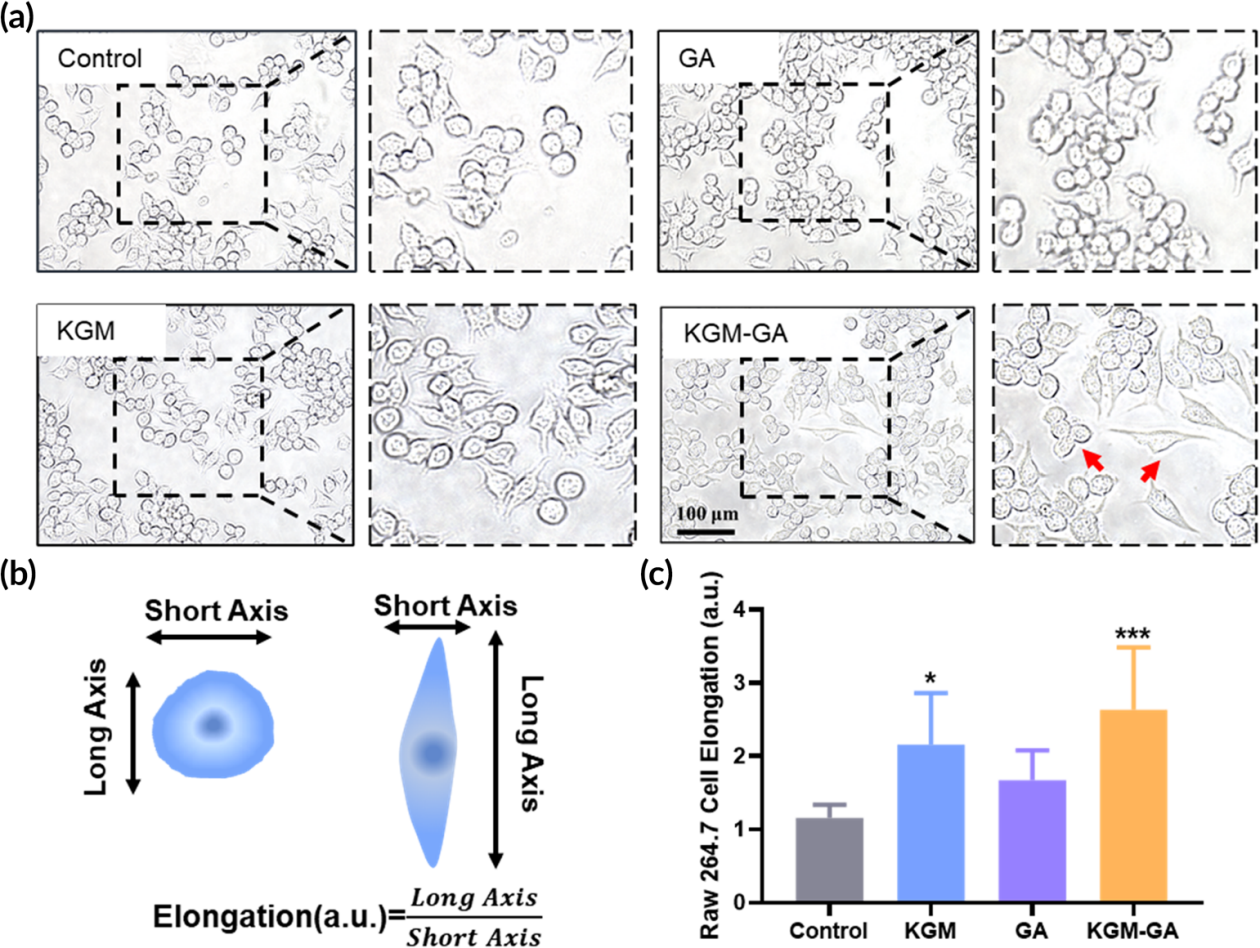
The morphological change of macrophages in vitro. (a) The morphology of Raw 264.7 cells was observed by optical microscope after 48 h exposure konjac glucomannan (KGM), gallic acid (GA), and KGM‐GA, respectively. Red arrowheads indicated round and elongated macrophages. (b) The Schematic of elongation calculation method. (c) Statistical data of the cell elongation change of Raw 264.7 macrophages. Tests were conducted by one‐way analysis of variance (ANOVA) with Tukey post hoc analysis. The data are presented as the mean ± SD (*n* = 10). ***p < 0.001, *p < 0.05 compared to control group

Morphological changes in macrophages are related to their functional polarization. Previous studies used different signals to stimulate morphological changes in macrophages from a round shape to an elongated shape, which tended to be M2 macrophages.^36^, ^37^ Based on the close relationships between the morphology and phenotype of macrophages, we first observed changes in Raw 264.7 macrophage morphology. Briefly, Raw 264.7 cells were incubated with KGM, GA, or KGM‐GA (50 μg/ml) solutions for 48 h, and cell morphology was captured with a microscope. Figure 2a shows that RAW 264.7 cells in the control group appeared round and contractive in morphology, and treatment with GA did not significantly change their morphology. In contrast, macrophages activated by KGM or KGM‐GA displayed elongated shapes and improved spreading morphology. A schematic diagram of cell morphological changes is shown in Figure 2b, and the formula for calculating elongation was described in previous research.^38^ As shown in Figure 2c, the control group exhibited an elongation of 1.15 ± 0.18, while the KGM and KGM‐GA groups exhibited elongations of 2.15 ± 0.7 and 2.77 ± 0.82, respectively, which were significantly different from those in the control group. As demonstrated by many studies, enhanced macrophage elongation is believed to be closely related to the M2 phenotype.

**FIGURE 3:**
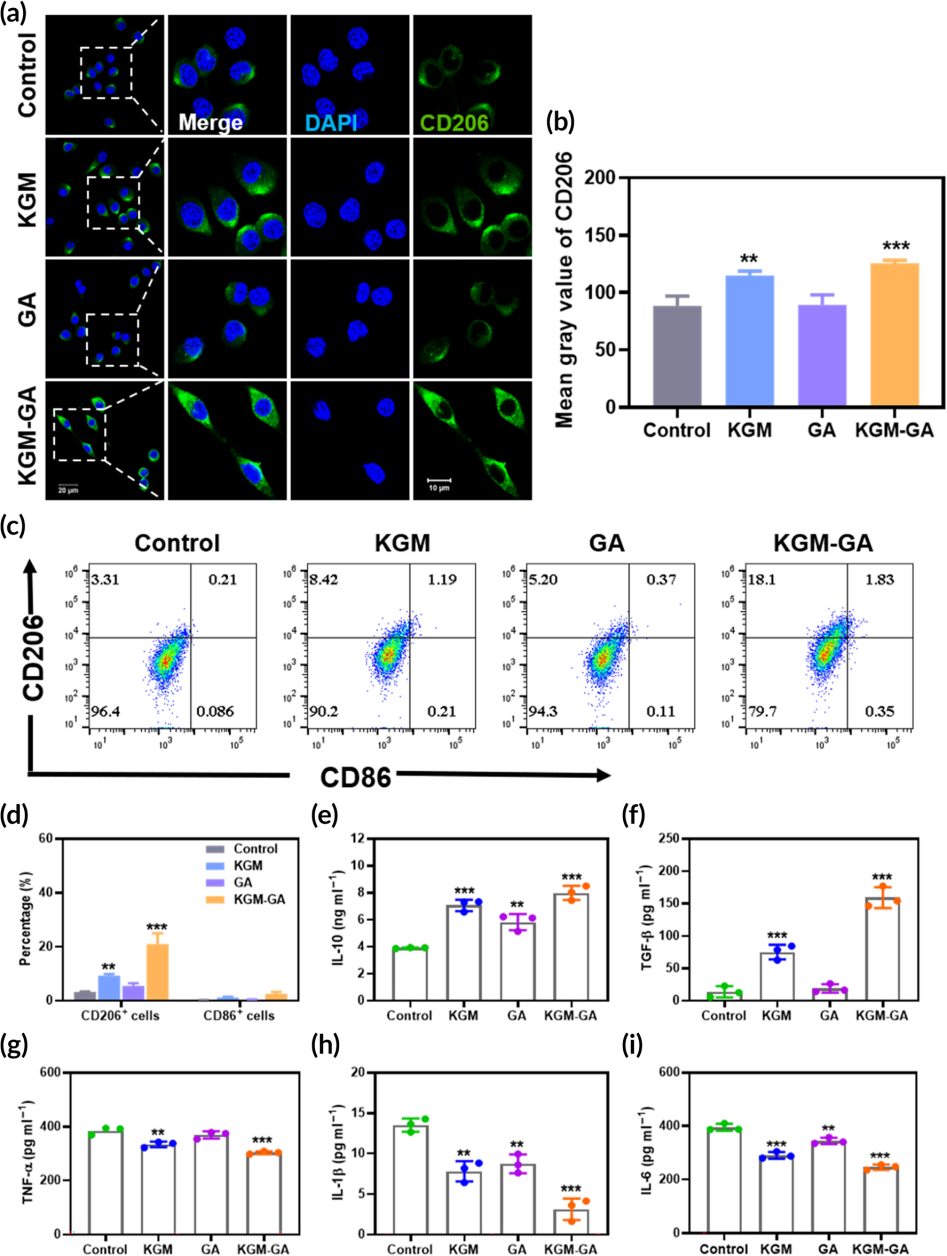
Konjac glucomannan–gallic acid (KGM‐GA) regulates polarization of macrophages in vitro. (a) Representative images of CD206 immunofluorescence staining of Raw 264.7 after 48 h cultured with KGM, GA, and KGM‐GA, respectively. (b) Quantitative measurement of CD206 signals. (c, d) Flow cytometry results of Raw 264.7 after cells were stained with M1 marker CD86 and M2 marker CD206. The ratios of M2 phenotypes were also presented. (e–i) The secretion of IL‐10, TGF‐β, tumor necrosis factor‐α (TNF‐α), interleukin‐1β (IL‐1β), and interleukin‐6 (IL‐6) in culture supernatants of Raw 264.7 cells stimulated by lipopolysaccharide (LPS). Tests were conducted by one‐way analysis of variance (ANOVA) with Tukey post hoc analysis. The data are presented as the mean ± SD (*n* = 3). ***p < 0.001, **p < 0.01, and *p < 0.05 compared to control group; ###*p* < 0.001 compared to KGM group

To further evaluate the effects of KGM‐GA on M2 polarization, we examined the expression of M1 and M2 macrophage markers. Immunofluorescence staining revealed that after 48 h of incubation with KGM‐GA, the expression of the M2 surface marker CD206 was significantly increased, and the intensity was greater than that induced by KGM (Figure 3a,b). In addition, these results suggested that KGM‐GA was a potent driver of M2 polarization without significantly stimulating the transformation of cells to the M1 phenotype. Quantitative analysis was followed by flow cytometry. As shown in Figure 3c,d, after KGM‐GA treatment, the percentage of cells expressing CD206 among total cells was 20.98% ± 4.1%, which was nearly 5.4 times higher than those in the control group, more than 3.8 times higher than those in the GA group, and approximately 2.2 times than those in the KGM group. In addition, we found that all groups expressed low levels of CD86, and there was no significant difference among the groups. Moreover, some cells coexpressed CD86 and CD206, indicating a transitional state between the M1 and M2 phenotypes in these macrophages. We examined the expression of M1 and M2 macrophage markers.

### 2.4 KGM‐GA inhibited the inflammatory response

To investigate whether KGM‐GA was involved in mediating the inflammatory effect of macrophages, Raw 264.7 cells were cultured with LPS to stimulate inflammation. After being treated with equivalent amounts of PBS, KGM, GA, and KGM‐GA, we measured the secretion of anti‐inflammatory IL‐10 and TGF‐β and proinflammatory tumor necrosis factor‐α (TNF‐α), interleukin‐1β (IL‐1β), and interleukin‐6 (IL‐6), by ELISA. As shown in Figure 3e, the secretion of IL‐10 in the KGM‐GA, KGM, and GA groups was significantly increased in comparison with that in the PBS group, and IL‐10 in the KGM‐GA group was also significantly enhanced compared with that in the KGM and GA groups. In addition, KGM and KGM‐GA significantly increased the secretion of TGF‐β by macrophages, which can be considered the result of the paracrine signaling in M2 macrophages (Figure 3f). TGF‐β promotes the proliferation and migration of fibroblasts and the production of ECM, which is beneficial in tissue repair. In contrast, KGM‐GA reduced the secretion of the proinflammatory cytokines TNF‐α, IL‐1β, and IL‐6 in comparison with that in the PBS group (Figure 3g–i). Although KGM and GA also inhibited the production of proinflammatory cytokines, the relative reduction in TNF‐α, IL‐1β, and IL‐6 in the KGM‐GA group was also significantly higher than that in the other groups. These results demonstrated that KGM‐GA inhibited the secretion of proinflammatory cytokines, enhanced anti‐inflammatory cytokine secretion in an LPS environment and exhibited potential in inflammatory‐related diseases.

### 2.5 ROS scavenging activities of KGM‐GA in vitro

**FIGURE 4:**
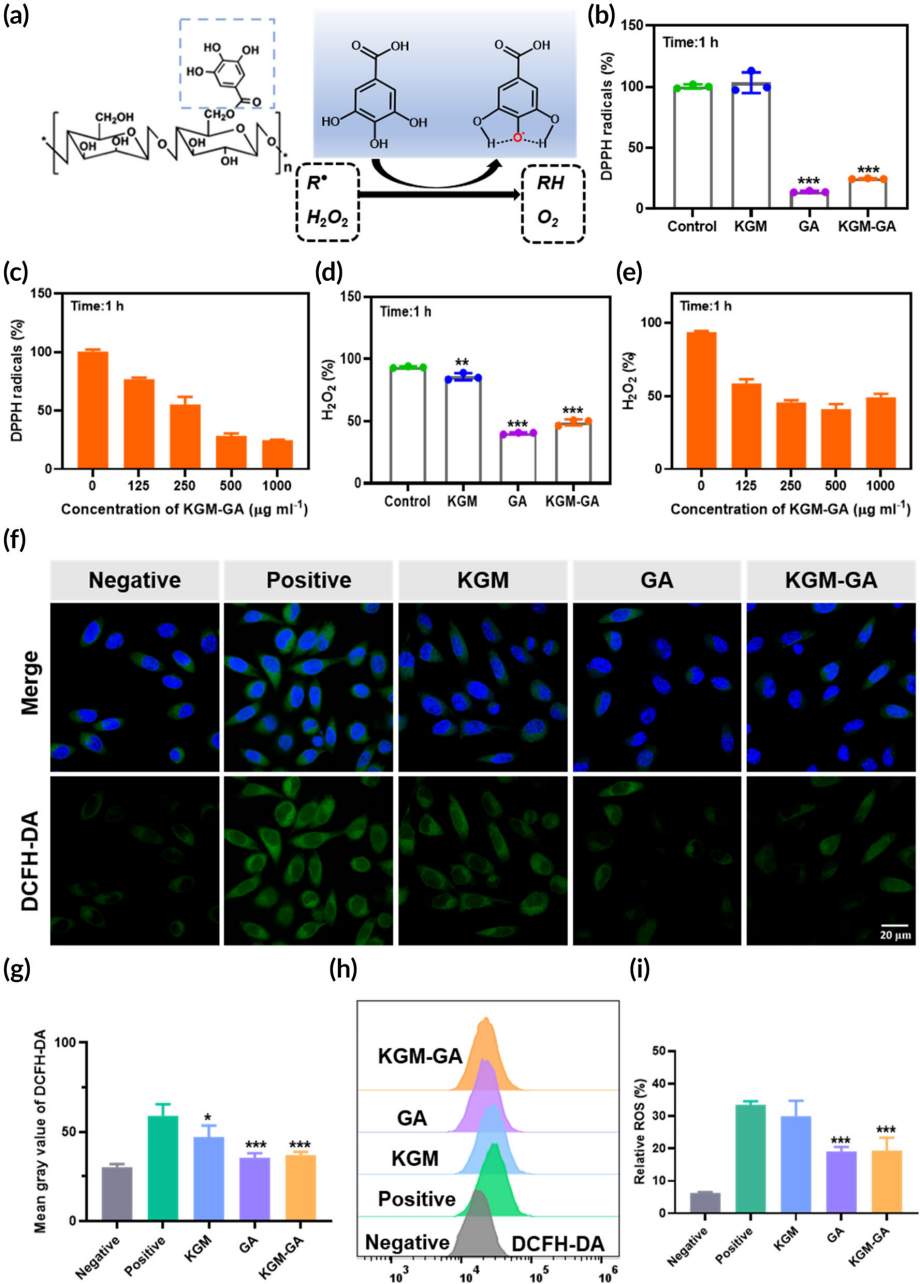
Reactive oxygen species (ROS) scavenging activities of konjac glucomannan–gallic acid (KGM‐GA) in vitro. (a) Schematic illustration of the ROS scavenging process with KGM‐GA. (b, c) Free‐radical scavenging ability of KGM, GA, and KGM‐GA. (d, e) H_2_O_2_ scavenging ability of KGM, GA and KGM‐GA. ***p < 0.001, **p < 0.01 compared to control group. (f) Confocal images of ROS levels in L929 cells using DCFH‐DA probe after incubation with 200 μM H_2_O_2_ and different treatments. (g) Quantitative analysis of fluorescence intensity of DCFH‐DA. (h, i) Statistical analysis of ROS levels in L929 cells under different treatment conditions by flow cytometer. Tests were conducted by one‐way ANOVA with Tukey post hoc analysis. The data are presented as the mean ± SD (*n* = 3). ***p < 0.001 compared to positive group

**FIGURE 5:**
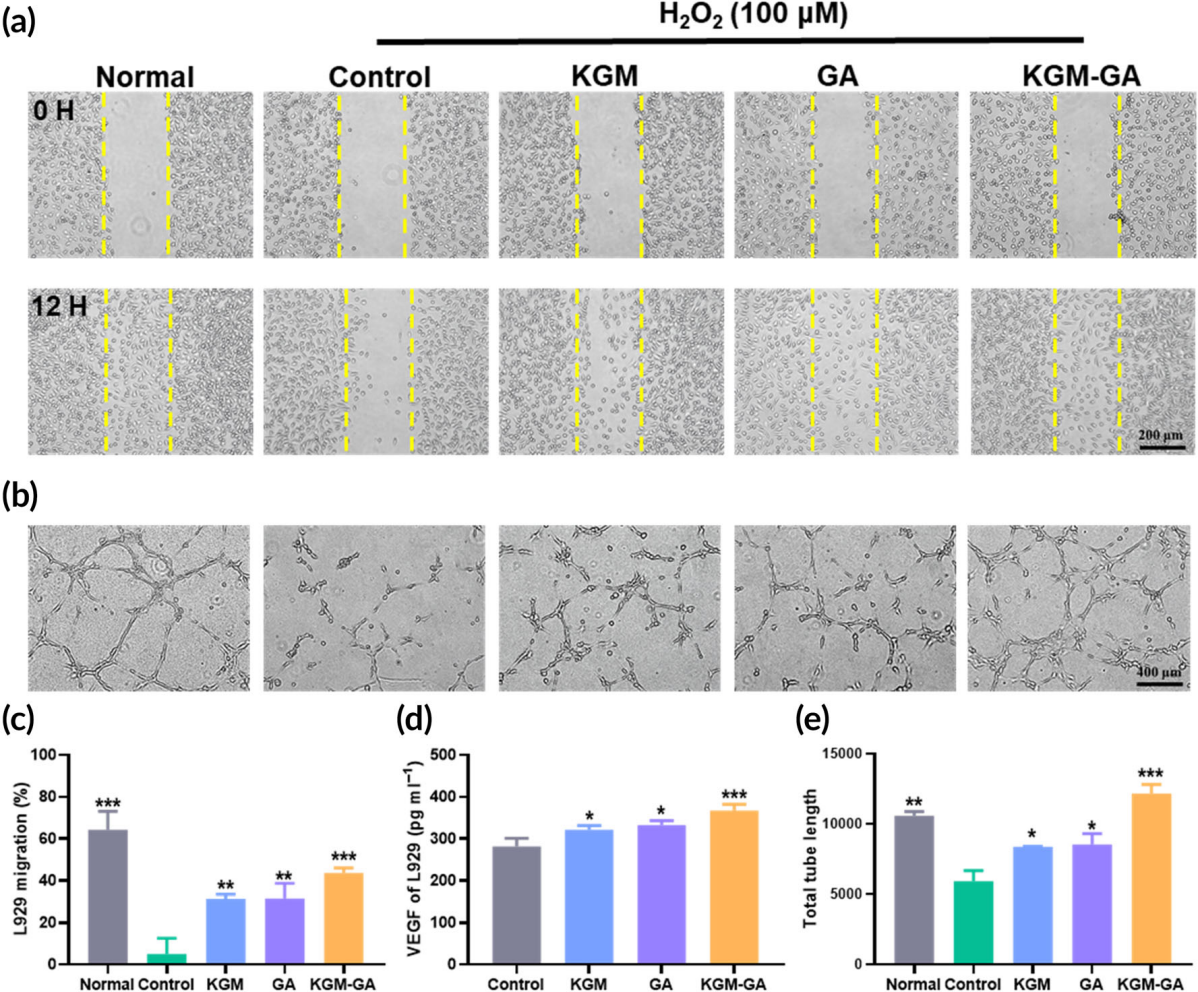
Effects of KGM‐GA on cell migration and vessel formation in vitro. (a) Cell migration images of L929 cells cultured with KGM, GA and KGM‐GA for 12 h after incubation with 100 μM H_2_O_2_. (b) Representative images of HUVECs tube formation after incubation with 100 μM H_2_O_2_ and different treatments. (c) Quantitative analysis of cell migration assay for L929 cells. (d) The level of VEGF in the culture supernatants of L929 cells treated with KGM, GA, and KGM‐GA for 48 h. (e) Quantitative analysis of tube formation for HUVECs. Tests were conducted by one‐way ANOVA with Tukey post hoc analysis. The data are presented as the mean ± SD (*n* = 3). ***p < 0.001, **p < 0.01, and *p < 0.05 compared to control group

The potential antioxidant activity of KGM‐GA was evaluated in this study (Figure 4a). Free radicals and H_2_O_2_ were selected as representative ROS to examine the ROS scavenging activities of KGM‐GA by DPPH and Amplex Red assays, respectively. As shown in Figure 4b, after being incubated with KGM for 1 h, almost no change was observed in the level of the DPPH radical. However, KGM‐GA showed dose‐dependent activity, the color of DPPH gradually changed from modena to yellow, and nearly 75% of DPPH was eliminated at the maximum concentration (1 mg/ml) (Figure 4c). Furthermore, the H_2_O_2_‐scavenging activity of KGM‐GA was studied. The concentration of H_2_O_2_ could be maintained at a low level, and more than 60% H_2_O_2_ was scavenged when the KGM‐GA concentration was 500 μg/ml compared with that in the control group (Figures 4d and 5e). Collectively, these results demonstrated that KGM failed to scavenge free radicals or H_2_O_2_ under the evaluated conditions. However, KGM‐GA showed amazing ROS scavenging activities, similar to that of GA due to the exposure of the polyphenol structure of GA.

Furthermore, we evaluated the ability of KGM‐GA to control intracellular ROS levels. In the positive control group, L929 cells were incubated with 200 μM H_2_O_2_ to induce oxidative stress damage and imitate the ROS environment, while cells cultured with medium alone served as the negative control. After being stimulated for 2 h, cells were treated with different interventions for an additional 2 h, and the intracellular ROS level was measured by a standard DCFH‐DA assay. As shown in Figure 4f,g, the fluorescent signals of DCFH‐DA were significantly increased in response to H_2_O_2,_ and KGM slightly reduced ROS levels. In comparison, when the cells were treated with GA or KGM‐GA, the intracellular ROS level obviously decreased, indicating that KGM‐GA could alleviate oxidative stress induced by H_2_O_2_. Moreover, the intracellular ROS levels were also quantitatively analyzed by flow cytometry (Figure 4h,i). The relative percentage ROS in L929 cells was reduced from nearly 35% to less than 20% after treatment with KGM‐GA. KGM slightly eliminated intracellular ROS levels in L929 cells, which may be related to KGM promoting the proliferation of fibroblasts by stimulating metabolism in cells, as reported.^17^ KGM was modified with GA, and KGM‐GA exerts an excellent effect against oxidative stress, which is highly suitable for wound healing and regeneration.

### 2.6 Cell migration and proangiogenic responses in vitro

Fibroblasts play a vital role in tissue repair. To evaluate the effect of KGM‐GA on fibroblasts, the migration of L929 cells was examined. As shown in Figure 5a, incubation with a certain concentration of H_2_O_2_ affected the migration of cells compared with those in the normal group. However, cells exposed to KGM, GA, and KGM‐GA showed a significant increase in migration compared with those in the control groups. After 12 h, cells exposed to KGM‐GA showed the highest migration (43.6% ± 2.4%), followed by those exposed to PBS (5.1% ± 7.3%), KGM (31.4% ± 2.0%), and GA (31.6% ± 7.1%) (Figure 5c). In addition, after being incubated with different materials for 48 h, the level of VEGF in the cell supernatant was measured. As shown in Figure 5d, KGM‐GA obviously improved the secretion of VEGF, suggesting that KGM‐GA promotes angiogenesis.

Since angiogenesis is a key process in skin tissue repair, we next evaluated the ability of KGM‐GA to drive angiogenesis in HUVECs. In a hydrogen peroxide environment, the vessel‐forming capability of HUVECs was examined by the tube formation assay. As shown in Figure 5b,e, in the normal group, the cells connected to form networks after 2 h of culture in Matrigel. However, under H_2_O_2_ conditions, the tube‐forming ability of HUVECs was seriously affected. Although sporadic tube formation was observed, the length was short, and the tubes did not form a dense network structure, indicating that the formation of blood vessels was blocked in the ROS environment. Furthermore, incubation with KGM, GA, and KGM‐GA enhanced tube formation in HUVECs in the ROS microenvironment. In particular, the effect of KGM‐GA was the most significant, and the cells formed junctions and compact tubes, as evidenced by increased branch lengths, which was not inferior to that of the normal group. Overall, KGM‐GA can significantly resist ROS produced by H_2_O_2_ to promote cell migration and vessel formation, indicating that KGM‐GA is capable of accelerating the wound healing process.

### 2.7 KGM‐GA improves wound healing in mice

**FIGURE 6:**
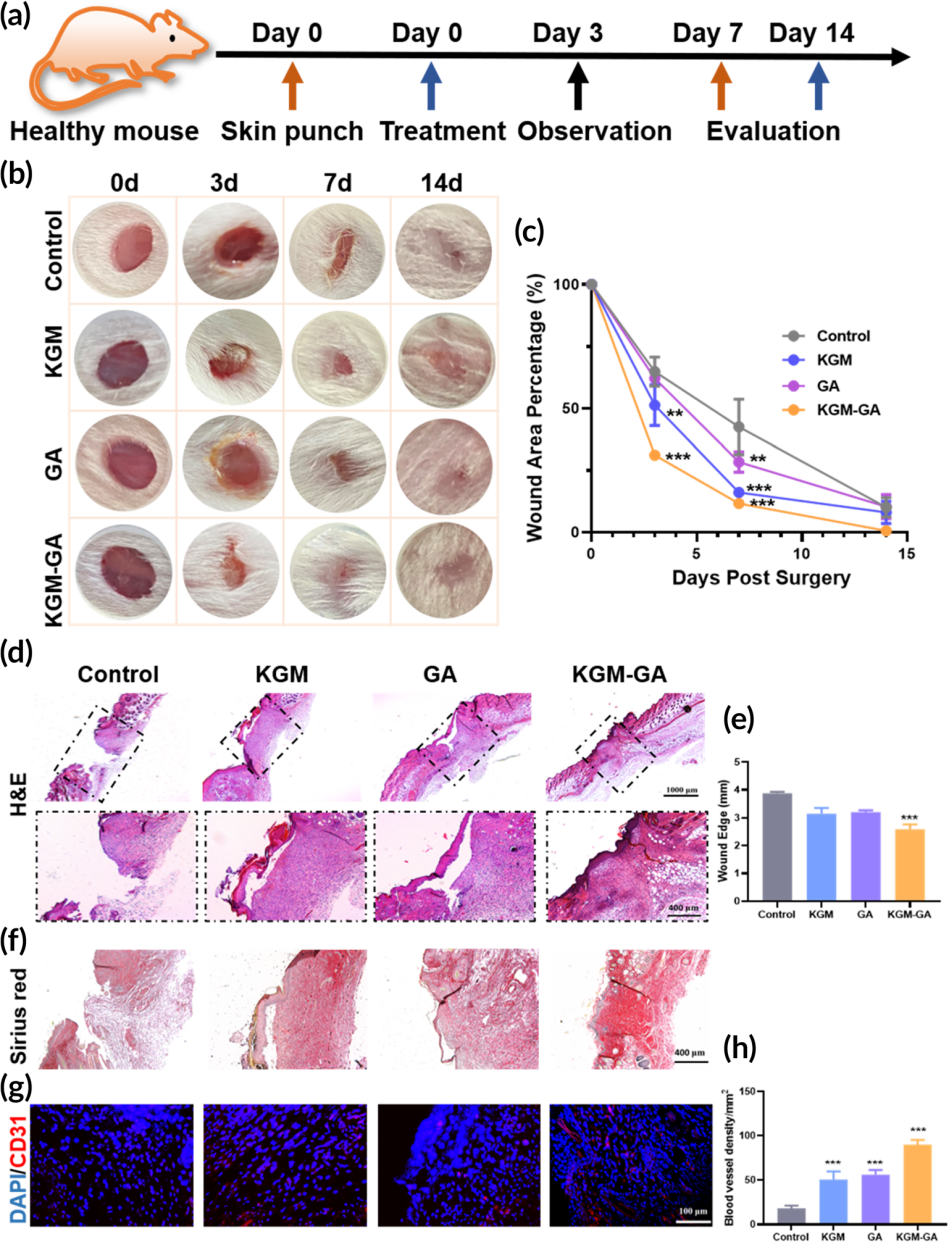
In vivo wound healing for mice under KGM‐GA treatment. (a) Scheme showing the process of the wounds treated by cultured with KGM, GA, and KGM‐GA. (b) Representative images of the wounds after treatment with various dressings at Days 0, 3, 7, and 14 post wounding. (c) Quantification of the process of wound area for all groups. (d, e) H&E staining of wound sections in all groups at Day 7. The note of the black dotted lines indicated the wound. Then, quantification of the process of wound edge for all groups. (F) Picrosirius Red staining of wound sections in all groups at Day 7. (g, h) Representative immunofluorescence data and the statistic results of CD31 stained sections at Day 14. Tests were conducted by one‐way ANOVA with Tukey post hoc analysis. The data are presented as the mean ± SD (*n* = 6). ***p < 0.001 and **p < 0.01 compared to control group

To evaluate the effect of KGM‐GA on wound healing, we created wounds on the backs of mice as in vivo models (Figure 6a). The wounds were treated with PBS, KGM, GA, and KGM‐GA, respectively, and images were captured at 0, 3, 7, and 14 days (Figure 6b,c). The images illustrated the progression of wound closure after treatment. Wounds in each group gradually decreased over time within 14 days, but the speed of wound closure in the KGM‐GA group was significantly faster than that in the other groups. At 3 days after the procedure, the wound area of KGM‐GA group (31.1% ± 0.85%) was significantly smaller than that of KGM (51.2% ± 8.22%), GA (62.1% ± 1.19%) and control group (64.8% ± 5.86%). After 7 or 14 days of treatment, the images further showed the best wound healing in the KGM‐GA group. To further assess the regeneration of skin tissues, we used H&E‐stained sections of healing areas for histological analysis of the wound (Figure 6d). First, on Day 7, the wound in the control group did not heal, with poor epithelialization and reduced granulation tissue formation. In contrast, the wound epidermis in the KGM‐GA group had formed, which exhibited a good therapeutic effect on granulation tissue. On Day 14, the control group showed a severe inflammatory response with the infiltration of inflammatory cells, while the epidermis, the dermis, sweat glands, and hair follicles appeared in the KGM‐GA group, indicating that the wound achieved complete skin regeneration (Figure S6). Furthermore, the average length of the wound edge was calculated and is shown in Figure 6e. The length in KGM‐GA group was significantly smaller than that in the control group (Figure 6d). In addition, the results of picrosirius red staining showed that collagen deposition in the KGM‐GA group was higher (red staining) and collagen fibers were arranged more regularly than in the control group (Figure 6f). CD31 is a vascular endothelial growth factor that can promote angiogenesis. An anti‐CD31 antibody was used to stain newly formed blood vessels in the wound. On Day 7, the count of capillary densities showed that a significantly higher density of capillaries was produced in KGM‐GA (Figure S7).

Moreover, after 14 days of treatment (Figure 6g,h), KGM‐GA promoted the expression of CD 31 and induced a higher density of capillaries (90.1 ± 5.3 capillaries per mm^2^) than in the control group (18.3 ± 3.0 capillaries per mm^2^), KGM group (56.1 ± 5.6 capillaries per mm^2^), or GA group (50.9 ± 9.2 capillaries per mm^2^). These results indicated that KGM‐GA effectively accelerated the wound healing process.

### 2.8 Regulation of the ROS microenvironment and macrophage phenotype in vivo

**FIGURE 7:**
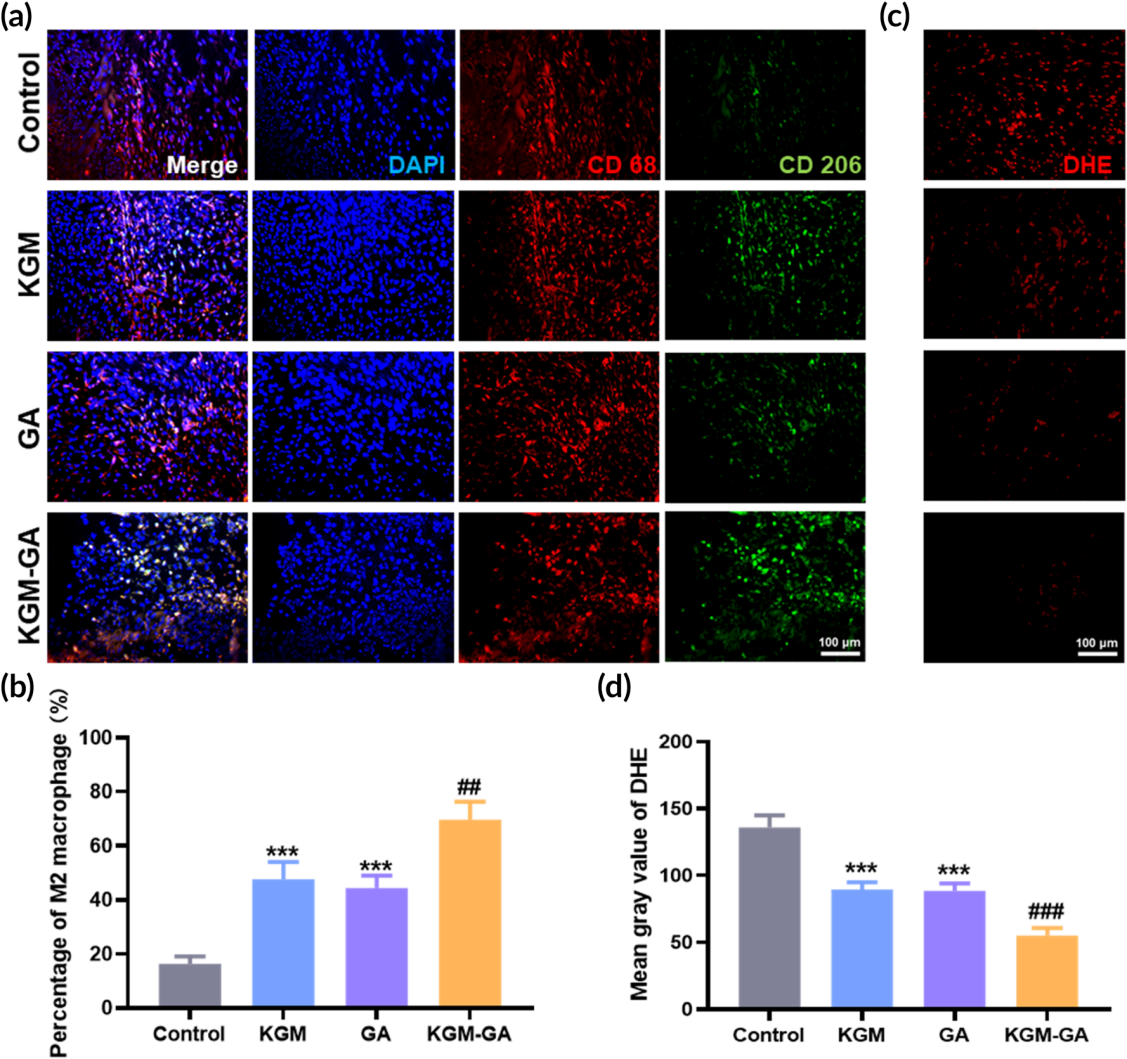
Konjac glucomannan–gallic acid (KGM‐GA) induced M2 macrophage polarization and alleviated reactive oxygen species (ROS) level. (a) Representative pictures of CD206/CD68 immunofluorescence staining of KGM, GA and KGM‐GA on Day 14 post wounding. (b) Statistical data of the percentage of M2 macrophages. (c) Representative pictures of DHE staining at Day 7 post wounding. (d) Statistical data of ROS levels in wound. Tests were conducted by one‐way ANOVA with Tukey post hoc analysis. The data are presented as the mean ± SD (*n* = 6). ***p < 0.001 compared to control group; ##*p* < 0.01 compared to GA group, ###*p* < 0.001 compared to KGM group

Based on the ability of KGM‐GA to eliminate ROS in vitro, we further investigated whether KGM‐GA could decrease oxidative stress in the skin wound healing model. After 7 days, the injured skin tissue was stained with DHE. From Figure 7c,d, abundant ROS were produced in the wound skin, which affected the healing of skin tissue, while GA and KGM‐GA treatment significantly reduced tissue ROS levels compared with KGM and PBS treatment. Next, we evaluated whether KGM‐GA could regulate macrophage polarization in vivo. On Day 14, the injured skin was collected for immunofluorescence staining to observe the expression of CD68 (a marker of all macrophage subsets) and CD206 (a marker of M2 macrophages) in the wound tissue. As shown in Figure 7a,b, in the subcutaneous layer of the wounds, the distribution of CD206‐positive cells in the KGM‐GA group was more widespread than that in the other groups and was approximately 4.2‐fold greater than that in the PBS group, indicating that KGM‐GA could stimulate macrophage polarization to the M2 phenotype in vivo.

## 3 DISCUSSION

The repair and regeneration of skin injuries caused by traumatic injury, surgery, or disease remain major clinical challenges and contribute to increased health care costs.^39^ Wound healing is a dynamic, intertwined, and complex process that includes multiple stages, and the prolongation of the inflammatory period adversely affects subsequent tissue regeneration.^40^ On the one hand, excessive inflammation at the damaged tissue site prolongs the inflammation period and delays the wound healing process, which may lead to the development of pathological fibrosis or scarring, thereby destroying normal tissue structure and function.^41^ On the other hand, delayed wound healing will greatly increase the risk of infection, which further aggravates the healing process and leads to a vicious cycle.^40^ Therefore, effective therapies for healing wounds targeting the inflammatory microenvironment of the injured site should be considered. It has been widely acknowledged that macrophages are important regulators of the wound healing process and are involved in advancing inflammation and promoting tissue repair and regeneration.^32^ Many studies have shown that the conversion of proinflammatory macrophages (M1 type) to an anti‐inflammatory phenotype (M2 type) is critical for normal wound repair and fibrosis reduction.^42^ In addition, inflammation is intimately associated with oxidative stress.^43^ An excessive inflammatory response at the wound site results in the production of a large amount of ROS,^44^, ^45^ which can aggravate local tissue damage, reduce the migration and proliferation of fibroblasts, keratinocytes, and endothelial cells, and delay wound healing.^46^

Recent studies have focused on unilateral regulation of macrophage polarization or the control of oxidative stress. A variety of biologically active substances have been used to modulate the phenotype of macrophages, including related cytokines,^47^, ^48^ receptors,^49^ small molecules,^50^ and mesenchymal stem cells.^10^, ^40^ However, their application is hindered because of uncontrollable biological activity. The use of biological materials to regulate cell fate and activity may lead to the development of new therapeutic strategies. Here, the natural polysaccharide KGM has attracted our attention due to its ability to regulate macrophage polarization and exposed chemical structures that are easily modified.^16^, ^51^ Recently, hydrolysates of KGM were shown to inhibit the production of proinflammatory cytokines and skew macrophage differentiation from M1 to M2 in the colon in mice with colitis.^51^ Furthermore, Jingjing Gan et al. prepared a type of KGM‐modified silica nanoparticle that could effectively induce MR aggregation on macrophages, thereby stimulating the cells to polarize to the M2 phenotype.^52^ Moreover, the polyphenol GA has been widely used due to its beneficial properties, including antioxidant and anti‐inflammatory properties. Previous studies have shown that polysaccharides are easily bound to other functional molecules, thus obtaining some properties, including antioxidant activity.^53^, ^54^ In this study, KGM was modified by GA through a simple and efficient technique to synthesize the wound dressing KGM‐GA, which exhibits antioxidant activity due to the reduction potential of hydroxyl groups in the aromatic structure of the phenolic ring. We also conducted in vitro and in vivo experiments to study the effect of KGM‐GA on the polarization of macrophages.

A wound dressing in the clinic is required to have excellent biocompatibility. The main components of KGM‐GA are two known natural products that exhibit good biocompatibility in vivo and in vitro, which is a prerequisite for acceptance and application, avoiding side effects caused by uncontrollable doses of biologically active molecules.^8^ More surprisingly, the combination of KGM‐GA did not weaken the biological activity, and the effect was better than that of KGM or GA alone. A possible explanation for this effect might be that the reaction was initiated by the hydroxyl group in KGM and the carboxyl group in GA. Thus, the groups that exerted the corresponding biological activity were not destroyed. Enhanced antioxidant properties and the ability of KGM‐GA to regulate the M2 phenotype were confirmed in subsequent experiments. In addition, fibroblast migration to and proliferation within the wound site are prerequisites for wound granulation during the proliferation stage, and angiogenesis is an indispensable step in the remodeling phase during wound healing.^55^ Excessive ROS accumulation inhibits fibroblast migration and angiogenesis, which was also confirmed in our experiments. Further statistical analysis revealed that KGM‐GA downregulated inflammation and improved oxidative stress to create an environment for the migration of L929 cells and tube formation by HUVECs.

Prior studies have shown that inherent contraction of skin wounds can only induce closure of the epidermis but cannot promote regeneration of the intact epidermal layer and mature dermis.^19^ This study confirmed that the application of KGM‐GA not only promoted wound healing but also improved skin tissue regeneration. Notably, KGM‐GA shortened the time required for wound closure. This effect may be related to the anti‐inflammatory effect of KGM‐GA on macrophages by promoting M2 polarization and inhibiting M1 polarization in the early stage of wound healing, shortening the transition time from the inflammation stage to the tissue formation stage. In addition, the low toxicity and antioxidant environment provided by KGM‐GA may help improve wound healing rates. In addition, the low toxicity and antioxidant environment provided by the GA component of KGM‐GA may help improve the wound healing rate. A relatively low level of ROS in the wound is considered to be the key to stimulating the release of cytokines, improving cell viability, and promoting blood vessel formation.^56^ Collectively, these results demonstrated that KGM‐GA could create a beneficial microenvironment for promoting tissue recovery and regeneration.

In most situations, wound repair will face unexpected challenges due to exposure to pathological conditions, such as aging, obesity, and diabetes. In diabetic wounds, macrophages exhibit a reduced capability to induce the phenotypic switch from M1 to M2 due to hyperglycemia and the presence of excessive glycosylation residues, resulting in accumulation and enrichment of M1 macrophages, causing excessive inflammation.^57^, ^58^ Therefore, wound healing in diabetes requires modulation of the polarization state of macrophages to reduce local inflammation. Currently, some studies have used polysaccharides to regulate macrophage phenotype to treat diabetic wound healing and have achieved certain therapeutic effects.^59^, ^60^, ^61^ In addition, excessive ROS accumulated in diabetic wounds hinders the regeneration of wound tissue.^19^, ^20^ Furthermore, in recent decades, inflammatory signals have not only been thought to influence wound healing but are also driving factors for diseases such as atherosclerosis^62^, ^63^ and cancer.^64^ The polymer we synthesized can regulate macrophages and combat oxidative stress without the aid of cytokine or gene delivery. The insights gained from this study may be of assistance in providing new strategies for the treatment of pathological wound injury and other inflammatory diseases.

## 4 MATERIALS AND METHODS

### 4.1 Materials

KGM was purchased from Karmachem (Shanghai, China). GA and 4‐dimethylaminopyridine (DMAP) were purchased from Heowns (Tianjin, China), 1‐ethyl‐3‐(3‐dimethylaminopropyl) carbodiimide hydrochloride (EDC. HCl) was obtained from Shanghai Medpep Co., Ltd. (Shanghai, China), and 2,7‐dichlorodihydrofluorescein diacetate (DCFH‐DA) was obtained from Sigma‐Aldrich (St. Louis, MO, USA). 1,1‐Diphenyl‐2‐picrylhydrazyl free radical (DPPH) was purchased from TCI (Shanghai, China). The CCK‐8 cell proliferation and cytotoxicity assay kit, live/dead cell staining kit and hematoxylin–eosin (HE) staining kit were purchased from Solarbio (Beijing, China). Dihydroethidium (DHE) was purchased from Beyotime (Shanghai, China). A Picrosirius Red staining kit was obtained from G‐CLONE (Beijing, China). Anti‐mouse ELISA kits for TNF‐α, IL‐6, IL‐1β, IL‐10, and TGF‐β and anti‐human ELISA kits for VEGF were obtained from Thermo Fisher Scientific (Beijing, China). Primary antibodies against CD68, CD206, and CD31 were purchased from Abcam (Cambridge, UK).

### 4.2 Cells and animals

The mouse L929 cell line, Raw264.7 cell line, and human umbilical vein endothelial cells (HUVECs) were obtained from the Cell Bank of Chinese Academy of Sciences. Female BALB/C mice were purchased from SPF (Beijing) Biotechnology Co. Ltd. (Beijing, China) (Approval No.: SCXK(Jing): 2019‐0010). All animal management procedures were reviewed and ethically approved by the Center of Tianjin Animal Experiment ethics committee and authority for animal protection (Tianjin, China). (License for use of experimental animals: approval No.: SYXK (Jin):2019‐0002).

### 4.3 Synthesis and characterization of KGM‐GA

KGM‐GA was synthesized via an esterification reaction according to a previously reported method. First, KGM (1 g) was dissolved in 200 ml of distilled water with magnetic stirring at 50°C until completely dissolved. Then, a mixture of GA (0.21 g, 1.11 mmol), EDC·HCl (0.355 g, 1.85 mmol), and DMAP (0.226 g, 1.85 mmol) was dissolved in distilled water (50 ml), and the solutions were stirred thoroughly for 0.5 h prior to the condensation reaction. Finally, the two solutions were completely mixed and reacted with stirring at 50°C for 72 h. After being cooled to room temperature, the reaction mixture was precipitated in absolute ethanol, repeatedly dissolved and precipitated three times. The final product (GA‐KGM) was obtained after drying at 60°C in a vacuum.

The chemical structure of GA‐KGM was confirmed by FTIR spectrometry (Thermo Fisher Scientific, Waltham, MA, USA). The content of GA in KGM‐GA was determined by measuring the absorbances of the GA and KGM‐GA solutions (0.5 mg/ml) at 263 nm by using a UV–vis spectrophotometer (PerkinElmer, USA). The morphology and structure of KGM‐GA were observed by scanning electron microscopy (SEM, ZEISS, Germany).

### 4.4 Cytotoxicity and blood compatibility assay

L929 cells and Raw 264.7 cells were used for cytology experiments. RPMI‐1640 medium containing 10% fetal bovine serum (FBS) was used for L929 cell maintenance, and Dulbecco's Modified Eagle Medium  containing 10% FBS was used for Raw264.7 cell maintenance. The cells were seeded in 96‐well plates (5000 cells/well) and incubated for 24 h at 37°C in a 5% CO_2_ atmosphere. Then, the cells were incubated with a series of concentrations of KGM, GA, and KGM‐GA for 24 h. Cell viability was evaluated by CCK‐8 cell proliferation and cytotoxicity assay kits (Solarbio, CA1210), and the absorbance at 450 nm was measured using a microplate reader (Thermo). Moreover, a live/dead cell staining kit (Solarbio, CA1630) was used to stain the cells. Finally, the images of the cells were observed by a fluorescence microscope from the Nikon Corporation (Tokyo, Japan).

The effect of KGM‐GA on erythrocyte hemolysis was examined according to a previously reported method.^65^ In brief, blood was collected from the orbital sinus and centrifuged at 3000 rpm for 15 min. Then, the erythrocytes were washed with PBS and treated with distilled water as a positive control, PBS as a negative control, or various concentrations of KGM‐GA for 2 h at 37°C. The samples were centrifuged at 3000 rpm for 15 min, and the absorbance of the supernatant at 540 nm was measured by a microplate reader.

### 4.5 KGM‐GA regulation of Raw 264.7 macrophage morphology

For morphological analysis, Raw 264.7 cells (1 × 10^5^ cells per well) were seeded in 24‐well plates and incubated overnight at 37°C, followed by treatment with KGM, GA, or KGM‐GA (50 μg/ml) solutions. After 48 h, the cells were washed with PBS three times to remove excess materials. The morphology of macrophages was captured by a microscope.

### 4.6 Immunofluorescence analysis of Raw 264.7 macrophages

For the in vitro macrophage polarization assay, Raw 264.7 cells (1 × 10^5^ cells per well) were seeded in confocal dishes and treated with or without KGM, GA, or KGM‐GA for 48 h at 37°C. Then, the cells were washed three times in PBS and fixed with 4% paraformaldehyde for 20 min. Subsequently, the cells were incubated with 5% bovine serum albumin (BSA) for 40 min at room temperature. After that, the cells were treated with 300 μl of a rabbit polyclonal antibody against CD206 (1:1000, Abcam, ab64693) diluted in PBS containing 1% BSA overnight in a wet box at 4°C. Then, the cells were washed with PBS and incubated with a goat polyclonal IgG FITC‐conjugated secondary antibody (1:100, HA1004, Huabio) diluted in PBS containing 1% BSA at room temperature. After 1 h, the cells were stained with DAPI and observed by a fluorescence microscope.

### 4.7 Flow cytometric analysis Raw 264.7 macrophages

Raw 264.7 cells were seeded in a 24‐well plate (1 × 10^5^ cells per well) in culture medium containing KGM, GA, or KGM‐GA for 48 h. After that, the cells were collected by centrifugation (1000 rpm, 5 min) and washed with PBS. Then, the cells were stained with PE‐conjugated anti‐mouse CD206 and FITC‐conjugated anti‐mouse CD86 antibodies for 20 min at 4°C and examined using a BD Accuri™ C6 flow cytometer.

### 4.8 ELISA analysis of proinflammatory and anti‐inflammatory factors

RAW264.7 cells were incubated in 24‐well plates (1 × 10^5^ cells per well) for 24 h and then stimulated with lipopolysaccharide (LPS, 2 μg/ml). After 24 h, the medium was removed, and fresh medium containing KGM, GA, or KGM‐GA was added and incubated for 48 h. Then, we collected the cell supernatants and measured the levels of IL‐1β, IL‐6, TNF‐α, interleukin‐10 (IL‐10), and transforming growth factor‐β (TGF‐β) by ELISA.

### 4.9 ROS‐scavenging ability evaluation

The H_2_O_2_ scavenging capacity of KGM‐GA was tested by the Amplex Red assay (Invitrogen, USA). Various concentrations of KGM, GA, or KGM‐GA (125–1000 μg/ml) were incubated with 40 μΜ H_2_O_2_, and the solution was left in a shaker (150 r/min) at 37 °C for 1 h. After the reaction, the concentration of the remaining H_2_O_2_ was determined according to the Amplex Red assay, and the H_2_O_2_‐scavenging ability was calculated.

In addition, to evaluate the free‐radical scavenging ability of KGM‐GA, a DPPH ethanol solution (200 μg/ml) was prepared. Subsequently, different concentrations of KGM, GA, or KGM‐GA (125–1000 μg/ml) were added to the same volume of DPPH solution and incubated for 1 h at 37°C. The absorption of the reacted solution at 515 nm was recorded by a microplate reader and used to calculate the free‐radical scavenging ability of KGM‐GA.

Furthermore, to study the intracellular ROS scavenging ability of KGM‐GA, L929 cells were seeded at a density of 1 × 10^5^ cells/well in confocal dishes for 24 h. Then, the cells were cultured with 200 μM H_2_O_2_ for 1 h. After being incubated, the culture media was removed and replaced with fresh media as a positive control or fresh media containing KGM, GA, or KGM‐GA (50 μg/ml). After 2 h, the cells were stained with 10 μM/L dichlorofluorescein diacetate (DCFH‐DA) for 30 min at 37°C. The fluorescence of the L929 cells was observed by a confocal fluorescence microscope from the Nikon Corporation (Tokyo, Japan). Meanwhile, L929 cells were seeded in 24‐well plates and incubated with H_2_O_2_ (200 μM) for 1 h and different treatments for 2 h. The cells were washed with PBS three times before the DCFH‐DA probe was added. The intracellular ROS level was evaluated by a BD Accuri™ C6 flow cytometer.

### 4.10 L929 cell migration and VEGF expression assay

The migration of L929 cells was evaluated. The cells were cultured in 24‐well plates (1 × 10^5^ cells/well) with FBS‐free medium for 24 h. Then, the cell monolayer was scratched in a straight line using a 200 μl pipette tip and washed with PBS to remove cell debris. After that, the cells were incubated with PBS, KGM, GA, or KGM‐GA containing 100 μM H_2_O_2_. Images of the scratched L929 cells were taken at 0 and 12 h and analyzed using ImageJ software. The cell migration rate was calculated as follows: Cell migration (%) = [(A0‐At)/A0] × 100%. A0 is the scratch area at 0 h, and At is the scratch area without cell migration at 12 h.

In addition, VEGF expression in L929 cells was measured by ELISA. In brief, the cells were seeded in 24‐well plates (1 × 10^5^ cells/well) and treated with KGM, GA, or KGM‐GA for 48 h. Finally, the supernatants were collected to measure VEGF levels.

### 4.11 HUVEC tube formation assay

For the tube formation assay, 100 μl of Matrigel (BD, USA) and 100 μl of medium were mixed, added to a precooled 48‐well plate and incubated at 37°C. After 30 min, HUVECs (3 × 10^4^ cells/well) were gently added to the culture plate and incubated with PBS, KGM, GA, or KGM‐GA containing 100 μM H_2_O_2_ for 4 h. Tube formation was captured by a microscope, and the total tube length was quantified using ImageJ software.

### 4.12 Wound healing in vivo

The therapeutic effect of KGM‐GA on wound healing was evaluated using a wound model. Female BALB/C mice (18–21 g, 6–7 weeks) were anesthetized by 4% chloral hydrate, and then the hair on the dorsal skin was shaved. Full‐thickness circular wounds with diameters of 6 mm were created by surgical procedures on the backs of the mice. The mice were randomly divided into four groups (*n* = 6) and treated with 50 μl of a saline solution dispersion of KGM, GA, or KGM‐GA (20 mg/ml) at the wound site. The other mice were treated with saline solution as controls. Images of the wounds were captured at Days 0, 3, 7, and 14 post‐wounding using a digital camera.

### 4.13 Histological analysis

After 7 and 14 days, the wound tissues were collected from the mice, fixed with 4% formaldehyde, and then embedded in paraffin. Then, the paraffin‐embedded tissues were sectioned into 6 μm thick slices for hematoxylin and eosin (H&E) and picrosirius red staining.

### 4.14 Immunofluorescence analysis

The skin sections were deparaffinized and rehydrated and then blocked with goat serum at room temperature for 30 min. The skin sections were treated at 4°C overnight with the following primary antibodies: CD68 mouse polyclonal antibody (1:200, Abcam, ab955), CD206 rabbit polyclonal antibody (1:200, Abcam, ab64693) or CD31 rabbit polyclonal antibody (1:50, Abcam, ab28364). Then, the sections were washed with PBS and incubated with Alexa Fluor 594 goat anti‐mouse IgG (1:500, Invitrogen, A11032) and FITC‐labeled goat anti‐rabbit IgG (1:200, Huabio, HA1004) or Cy3‐labeled goat anti‐rabbit IgG (1:200, Beyotime, A0516) and secondary antibodies at room temperature for 2 h. Finally, the sections were counterstained with DAPI and mounted with antifade mounting medium. Fluorescence images were captured by a fluorescence microscope.

### 4.15 ROS measurement in wound sites

The wound specimens at Day 7 post‐wounding were washed with PBS three times. After that, the skin sections were incubated with 2.5 μM dihydroethidium (DHE) at 37°C for 30 min and imaged with a fluorescence microscope.

### 4.16 In vivo biocompatibility of KGM‐GA

At 14 days of treatment, blood samples were collected for complete blood panel analysis. In addition, major organs, including the heart, liver, spleen, lung, and kidney, were removed, and paraffin sections were prepared. HE staining was performed to assess tissue structure. All data are compared with normal mice.

### 4.17 Statistical analysis

The data are presented as the mean ± standard deviation. Where appropriate, a one‐way or two‐way analysis of variance (ANOVA) was performed to assess statistical significance. Statistical evaluations were performed using GraphPad Prism 8.0, and *p* values <0.05 were considered statistically significant.

## 5 CONCLUSION

In this study, we present a simple and efficient technique to synthesize a polymer with good biocompatibility. KGM‐GA exhibited obvious antioxidant properties and the ability to regulate M2 macrophages, thereby inhibiting the secretion of inflammatory factors in vitro. In a mouse model of full‐thickness skin injury, KGM‐GA alleviated oxidative stress and the inflammatory response at the wound site, accelerated wound closure and collagen deposition and enhanced angiogenesis, leading to rapid skin regeneration. Our results suggest that KGM‐GA is an exceptionally meaningful and promising agent for wound healing.

## AUTHOR CONTRIBUTIONS

**Huiyang Li:** Data curation (lead); formal analysis (lead); investigation (equal); methodology (equal); validation (equal); writing – original draft (equal). **Xiaoyu Liang:** Investigation (equal); writing – review and editing (supporting). **Youlu Chen:** Writing – review and editing (supporting). **Kaijing Liu:** Writing – review and editing (supporting). **Xue Fu:** Writing – review and editing (supporting). **Xiaoli Wang:** Methodology (equal). **Jing Yang:** Conceptualization (equal); funding acquisition (equal); methodology (lead); project administration (lead); supervision (equal); writing – review and editing (lead). **Chuangnian Zhang:** Conceptualization (equal); funding acquisition (equal); methodology (lead); supervision (equal).

## CONFLICT OF INTEREST

The authors declare that they have no known competing financial interests or personal relationships that could have appeared to influence the work reported in this paper.

## Supporting information


**Figure S1** Viability of L929 cells treated with KGM, GA, and KGM‐GA, respectively, for 24 h at different concentrations.
**Figure S2.** Live/dead cell staining assay to examine the viability of L929 cells (A‐B) and Raw 264.7 (C‐D) treated with KGM‐GA for 24 h at different concentrations. Tests were conducted by one‐way ANOVA with Tukey post hoc analysis.
**Figure S3.** Evaluate the effect of KGM‐GA at different concentrations on the hemolysis of red blood cells.
**Figure S4.** Raw 264.7 cells were treated with different KGM‐GA at different concentrations for 48 h, flow cytometric analysis of the expression of CD206 and CD86 on cells.
**Figure S5.** Evaluate the ratio of CD206 to CD86 on Raw 264.7 cells. Tests were conducted by one‐way ANOVA with Tukey post hoc analysis. The data are presented as the mean ± SD (*n* = 3). ***p < 0.001 and *p < 0.05 compared to control group.
**Figure S6.** H&E staining of wound sections in all groups at Day 14. The note of the orange lines indicated the wound.
**Figure S7.** Representative immunofluorescence data and the statistic results of CD31 stained sections at Day 7. Tests were conducted by one‐way ANOVA with Tukey post hoc analysis. The data are presented as the mean ± SD (*n* = 6). ***p < 0.001 compared to control group.

## Data Availability

Data available on request from the authors.
