# Supplementary material for: Synergy of antioxidant and M2 polarization in polyphenol‐modified konjac glucomannan dressing for remodeling wound healing microenvironment
Source: Bioeng Transl Med. 2022 Sep 5;8(2):e10398. doi: 10.1002/btm2.10398 (PMC10013815; doi:10.1002/btm2.10398)
Supplement: Supplementary file 1 — Figure S1 Viability of L929 cells treated with KGM, GA, and KGM‐GA, respectively, for 24 h at different concentrations. Figure S2. Live/dead cell staining assay to examine the viability of L929 cells (A‐B) and Raw 264.7 (C‐D) treated with KGM‐GA for 24 h at different concentrations. Tests were conducted by one‐way ANOVA with Tukey post hoc analysis. Figure S3. Evaluate the effect of KGM‐GA at different concentrations on the hemolysis of red blood cells. Figure S4. Raw 264.7 cells were treated with different KGM‐GA at different concentrations for 48 h, flow cytometric analysis of the expression of CD206 and CD86 on cells. Figure S5. Evaluate the ratio of CD206 to CD86 on Raw 264.7 cells. Tests were conducted by one‐way ANOVA with Tukey post hoc analysis. The data are presented as the mean ± SD (n = 3). ***p < 0.001 and *p < 0.05 compared to control group. Figure S6. H&E staining of wound sections in all groups at Day 14. The note of the orange lines indicated the wound. Figure S7. Representative immunofluorescence data and the statistic results of CD31 stained sections at Day 7. Tests were conducted by one‐way ANOVA with Tukey post hoc analysis. The data are presented as the mean ± SD (n = 6). ***p < 0.001 compared to control group. [file BTM2-8-e10398-s001.docx]

**Supplementary Information**

**Synergy of antioxidant and M2 polarization in polyphenol-modified konjac glucomannan dressing for remodeling wound healing microenvironment**

Huiyang Li ^a^, Xiaoyu Liang ^a^, Youlu Chen ^a^, Kaijing Liu ^a^, Xue Fu ^a^, Chuangnian Zhang ^a *^, Xiaoli Wang ^a^, Jing Yang ^a*^

^a^ *Tianjin Key Laboratory of Biomaterial Research, Institute of Biomedical Engineering, Chinese Academy of Medical Science & Peking Union Medical College, Tianjin, 300192, China*

* Corresponding authors.

*E-mail addresses:* [cnzhang@mail.nankai.edu.cn](mailto:cnzhang@mail.nankai.edu.cn) (Chuangnian Zhang, PhD)

[yangjing37@hotmail.com](mailto:yangjing37@hotmail.com) (Jing Yang, PhD)

**Supplementary Figures**

**Figure S1.** Viability of L929 cells treated with KGM, GA, and KGM-GA, respectively, for 24 h at different concentrations.


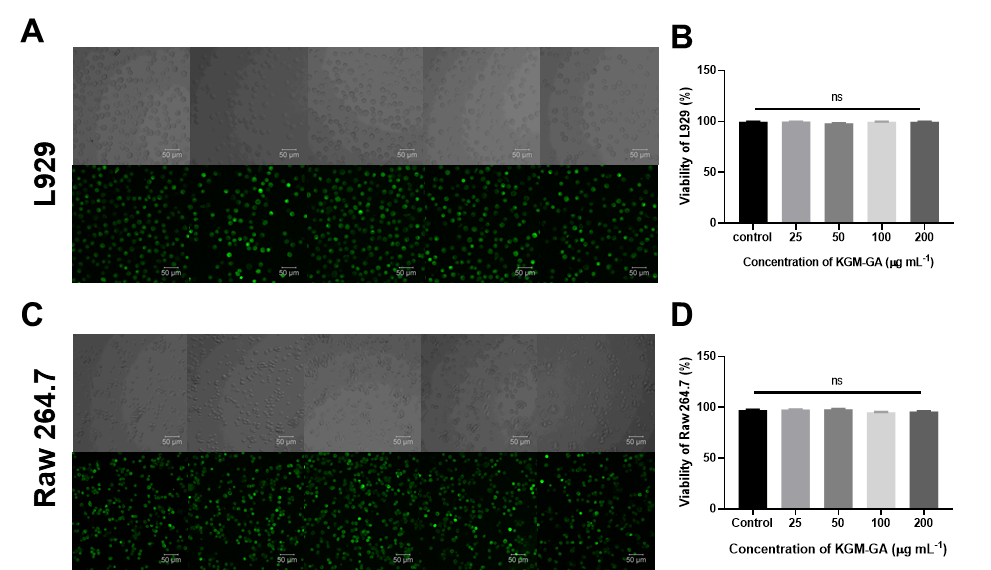


**Figure S2.** Live/dead cell staining assay to examine the viability of L929 cells (A-B) and Raw 264.7 (C-D) treated with KGM-GA for 24 h at different concentrations. Tests were conducted by one-way ANOVA with Tukey post hoc analysis.


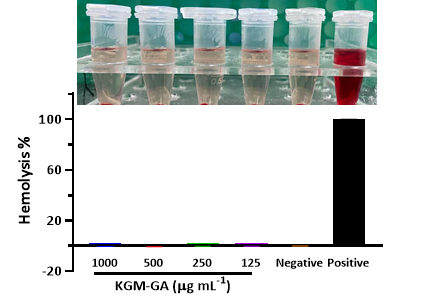


**Figure S3.** Evaluate the effect of KGM-GA at different concentrations on the hemolysis of red blood cells.

**Figure S4.** Raw 264.7 cells were treated with different KGM-GA at different concentrations for 48 h, flow cytometric analysis of the expression of CD206 and CD86 on cells.

**Figure S5.** Evaluate the ratio of CD206 to CD86 on Raw 264.7 cells. Tests were conducted by one-way ANOVA with Tukey post hoc analysis. The data are presented as the mean ± SD (n=3). ***P < 0.001 and *P < 0.05 compared to control group.


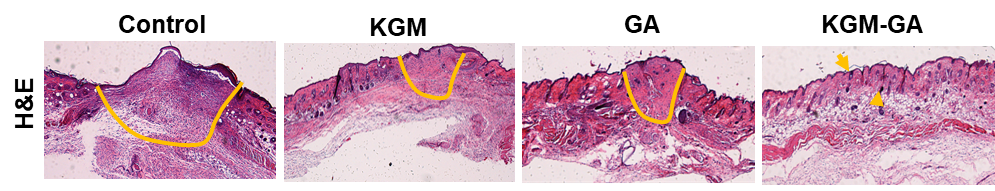


**Figure S6.** H&E staining of wound sections in all groups at day 14. The note of the orange lines indicated the wound.


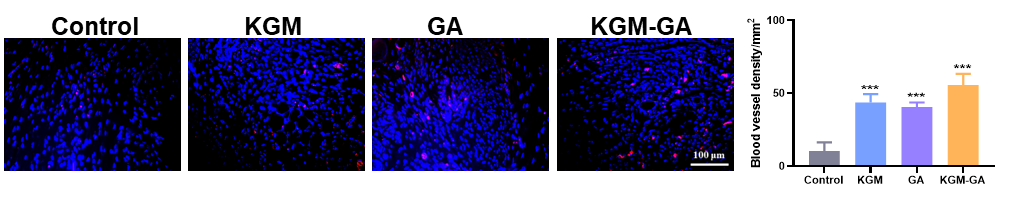


**Figure S7.** Representative immunofluorescence data and the statistic results of CD31 stained sections at day 7. Tests were conducted by one-way ANOVA with Tukey post hoc analysis. The data are presented as the mean ± SD (n=6). ***P < 0.001 compared to control group.
